# Machine learning enhanced evaluation of semiconductor quantum dots

**DOI:** 10.1038/s41598-024-54615-7

**Published:** 2024-02-20

**Authors:** Emilio Corcione, Fabian Jakob, Lukas Wagner, Raphael Joos, Andre Bisquerra, Marcel Schmidt, Andreas D. Wieck, Arne Ludwig, Michael Jetter, Simone L. Portalupi, Peter Michler, Cristina Tarín

**Affiliations:** 1 Institute for System Dynamics, University of Stuttgart, Stuttgart, Germany; 2 Institut für Halbleiteroptik und Funktionelle Grenzflächen, University of Stuttgart, Stuttgart, Germany; 3 Research Center SCoPE, University of Stuttgart, Stuttgart, Germany; 4 Center for Integrated Quantum Science and Technology, University of Stuttgart, Stuttgart, Germany; 5 Munich Institute of Robotics and System Intelligence, Technical University of Munich, Munich, Germany; 6 Lehrstuhl für Angewandte Festkörperphysik, Ruhr Universität Bochum, Bochum, Germany

**Keywords:** Quantum technology, Semiconductor quantum dot, Single photon source, Machine-learning-based evaluation, Convolutional autoencoder, Neural network regression, Computational science, Scientific data, Photonic devices, Quantum optics, Single photons and quantum effects, Applied physics, Computational science, Scientific data, Photonic devices, Quantum optics, Single photons and quantum effects, Applied physics

## Abstract

A key challenge in quantum photonics today is the efficient and on-demand generation of high-quality single photons and entangled photon pairs. In this regard, one of the most promising types of emitters are semiconductor quantum dots, fluorescent nanostructures also described as artificial atoms. The main technological challenge in upscaling to an industrial level is the typically random spatial and spectral distribution in their growth. Furthermore, depending on the intended application, different requirements are imposed on a quantum dot, which are reflected in its spectral properties. Given that an in-depth suitability analysis is lengthy and costly, it is common practice to pre-select promising candidate quantum dots using their emission spectrum. Currently, this is done by hand. Therefore, to automate and expedite this process, in this paper, we propose a data-driven machine-learning-based method of evaluating the applicability of a semiconductor quantum dot as single photon source. For this, first, a minimally redundant, but maximally relevant feature representation for quantum dot emission spectra is derived by combining conventional spectral analysis with an autoencoding convolutional neural network. The obtained feature vector is subsequently used as input to a neural network regression model, which is specifically designed to not only return a rating score, gauging the technical suitability of a quantum dot, but also a measure of confidence for its evaluation. For training and testing, a large dataset of self-assembled InAs/GaAs semiconductor quantum dot emission spectra is used, partially labelled by a team of experts in the field. Overall, highly convincing results are achieved, as quantum dots are reliably evaluated correctly. Note, that the presented methodology can account for different spectral requirements and is applicable regardless of the underlying photonic structure, fabrication method and material composition. We therefore consider it the first step towards a fully integrated evaluation framework for quantum dots, proving the use of machine learning beneficial in the advancement of future quantum technologies.

## Introduction

Advances in fundamental research and engineering over the past years have enabled the active control of systems within the framework of quantum mechanics, leading to the emergence of next generation quantum technologies. Development in this field is motivated by two main aspects: On the one hand, the progressive miniaturisation of devices down to the nanoscale inevitably requires the explicit consideration of quantum effects^1,2^. On the other hand, in some areas, a superior performance is expected, for instance in metrology, where sensors based on quantum principles offer a significantly higher sensitivity^3,4^. At current, efforts focus on the transition from proof-of-concept laboratory applications to commercially available products^5,6^.

One promising branch of quantum technology is applied quantum optics, or photonics, which is built around the single photon, i.e. the elementary particle of light^7,8^. In general, photonic solutions are attractive since photons provide several degrees of freedom to encode information and combine high mobility with intrinsic robustness against decoherence and environmental noise. This makes them particularly advantageous, e.g. for long-distance communication through optical fibres^9,10^. Besides, photons are comparatively easy to manipulate, making photonic setups experimentally very accessible^11,12^. Obviously, the development of an efficient and on-demand single photon source is key, with brightness, purity and indistinguishability of the emitted photons taking priority^13,14^. Many setups today make use of spontaneous parametric down-conversion, where a pair of entangled photons is generated from laser light in a non-linear birefringent crystal. While photons produced this way are highly indistinguishable, there is an intrinsic trade-off between brightness and single photon purity due to the Poissonian statistics of down-conversion^15,16^. Improving on these limiting factors, quantum light sources embedding semiconductor quantum dots in photonic structures or cavity resonators have increasingly established themselves as promising candidates and valid alternative^17,18^.

### Semiconductor quantum dots

**Figure 1:**
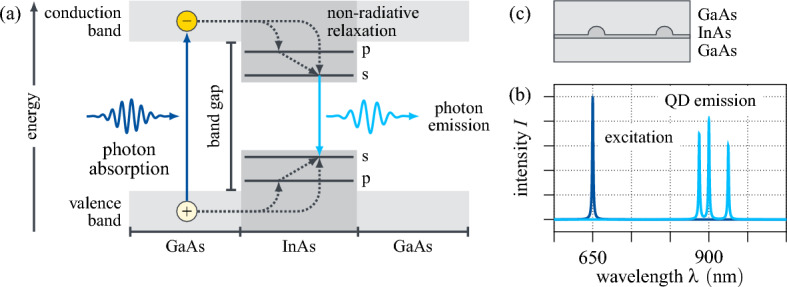
(**a**) Visual abstract of the fluorescent single photon emission of semiconductor QDs. Above-band excitation promotes an electron to the conduction band, from where it non-radiatively relaxes to the s-shell in the QD. The electron subsequently recombines with the electron hole left behind in the valence band and thereby emits a single photon with the energy of the QD state. (**b**) Schematic spectrum of the excitation laser (dark blue) and the QD emission (light blue). (**c**) Schematic side view of a ${\text {InAs/GaAs}}$ QD sample formed through epitaxial self-assembly.

Semiconductor quantum dots (QD) are nanoscale heterostructures with a lower band gap between the disjoint valence and the conduction band than their semiconductor environment. Their small size in terms of the de Broglie wavelength of electrons confines charge carriers (electrons, holes) in all three spatial dimensions, which results in a band structure allowing for discretised, i.e. quantised electronic states resembling the shells of atoms^13,19^. Their energetic landscape is graphically outlined in depth in Fig. 1a. Here, photons are represented as blue wavelets, while solid arrows trace optical transitions and dotted arrows indicate non-radiative relaxation. As shown, under above-band laser irradiation (dark blue), an electron is promoted from the low-energy valence band to the high-energy conduction band by absorbing a photon whose energy exceeds the band gap. Through non-radiative energy dissipation, the excited electron and the remaining hole relax to the respective lowest energy state of the QD (s-shell), forming a bound pair called exciton. This subsequently fluorescently recombines, i.e. the electron radiatively decays by emitting a single photon with the energy of the occupied QD state (light blue). The fluorescence wavelength is dependent on the quantisation of the QD states, which, in return, is directly determined by the size and geometry of the QD^20,21^.

Interestingly, additional charge carriers can typically be found in a QD, causing real QD spectra to exhibit multiple emission lines. This is showcased in Fig. 1b, where the spectra of the excitation laser (dark blue) and the QD emission (light blue) are schematically plotted as functions of the photon wavelength $\lambda$.

**Figure 2:**
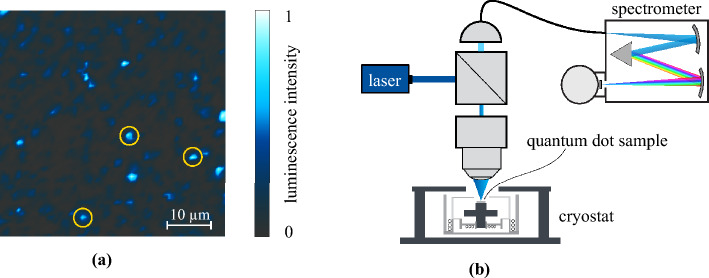
Confocal $\mu$-photoluminescence intensity measurement. (**a**) Normalised emission intensity in a 50×50 μm^2^ area of a representative self-assembled InAs/GaAs QD sample. Three exemplary QDs are marked in yellow. (**b**) Schematic measurement setup: the QD sample is placed inside a Helium cryostat and excited by an above-band laser guided through a beamsplitter. The luminescence signal is collected and sent to a spectrometer.

**Figure 3:**
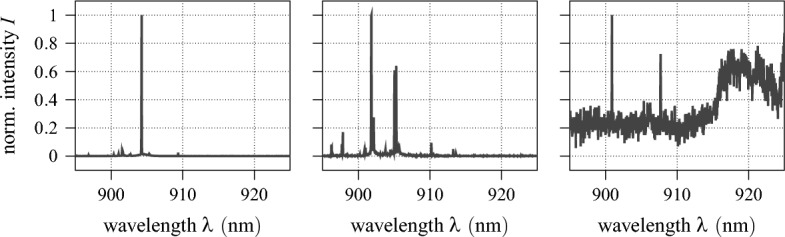
Emission spectra of the QDs encircled in Fig. 2a from left to right. The spectra are recorded between ${895}\,{\textrm{nm}}$ and ${925}\,{\textrm{nm}}$ wavelength in ${1024}\,{\textrm{px}}$ and normalised independently.

While there are several fabrication techniques and material compositions for the realisation of QDs, for this work, we consider self-assembled ${\text {InAs}}$ QD samples grown in the Stranski–Krastanow mode on a ${\text {GaAs}}$ platform using molecular beam epitaxy^22^. A schematic cross-section of such an ${\text {InAs/GaAs}}$ QD wafer is given in Fig. 1c. Despite having state-of-the-art performance in their photon emission properties, as a result of self-assembly, these QDs grow randomly distributed in space. On top of that, even when synthesised under the same growth conditions, their size can vary from dot to dot, resulting in different quantum confinement and therefore different emission wavelengths^23^. This implies, that in order to perform some intended experiment, a suitable QD has to be found on a given wafer. This is exemplarily illustrated in Fig. 2a, where a confocal $\mu$-photoluminescence intensity map^24^ of a representative ${\text {InAs/GaAs}}$ wafer is shown. The image is recorded using the measurement setup schematised in Fig. 2b: an above-band laser is scanned across the sample in a $50\times 50\,{\upmu }\textrm{m}^{2}$ area, while the QD luminescence is collected and analysed in a spectrometer. Plotting the intensity over the spatial position yields the colour map on the left, where QDs appear as bright spots over a dark background. The normalised emission spectra $I\left( \lambda \right)$ of the yellow encircled QDs are given in Fig. 3. All three emit at a fluorescence wavelength of about ${900}\,{\textrm{nm}}$. However, while in the first spectrum one optical transition is predominant, in the second one, in contrast, several transitions are excited simultaneously, resulting in the spectrum having multiple peaks. The third one, finally, besides having a significantly worse signal-to-noise ratio and an elevated baseline, exhibits a broadband feature in its emission spectrum at around ${920}\,{\textrm{nm}}$. For use as single photon sources in photonic systems, QDs ideally emit at only one specific, spectrally isolated wavelength with high intensity. In this regard, only the first QD appears to be applicable. Nevertheless, this does not mean that the other two are to be discarded straight away. For instance, with more sophisticated excitation schemes a single desired optical transition can be addressed. Equally, spectrally matching a photonic resonator to the driven transition and exploiting cavity quantum electrodynamic effects can prove advantageous^25,26^. Then again, a seemingly perfect QD photon source can turn out to be unfit on closer inspection, e.g. in a polarisation measurement^27^ or a surface topography^28^. Overall, this implies the suitability of a semiconductor QD as single photon source cannot be definitively decided solely based on its emission spectrum, but that further analyses are necessary. However, given that these are usually either time-consuming and/or resource expensive promising candidates must be pre-selected. Currently, this is done by hand: specifically trained experts consider the emission spectra of all candidate QDs on a given sample and assess based on their subjective experience whether they meet the spectral requirements for additional in-depth investigations and an eventual application. This evaluation is neither trivial, nor are there any quantified conventions for it. Both in research and industrial applications, this manual selection process represents an actual bottleneck limiting the productivity and thus the scalability of photonic technologies.

### Contribution

This paper directly addresses this challenge. Here, we propose a data-driven machine-learning-based solution for the automated evaluation of the applicability of a semiconductor QD as single photon source based on its emission spectrum. Specifically, the goal is to build an expert system approximating the current experience-based suitability analysis. With this contribution, the (pre-)selection of viable QDs can be parallelised and processing times can be significantly reduced. Moreover, the technical relevance of this paper increases in the long-term, as methods like the one presented here are a necessary requirement for a large scale production of semiconductor QD single photon sources and thus for an industrial implementation of photonic technologies. To the authors’ best knowledge, at the time of writing, no such approach has yet been reported in the literature. In Ref.^29^, a machine-learning-based classifier of quantum sources is proposed, although it is limited to discriminating between the emission of single and non-single photons from nitrogen-vacancy centres in diamond. Otherwise, the focus lies on using machine learning to enhance the QD fabrication process itself^30,31^. In particular, a variety of methods is employed to either provide the design parameters of the synthesis^32,33^ or to make predictions about the resulting optical properties^34,35^.

This paper is structured as follows: after covering the physical background and relevance of the topic in the introduction, next, the evaluation algorithm is presented in depth and contextualized within the state of the art. It is subsequently implemented and its performance showcased, which, finally, allows for a discussion of the proposed solution.

## Quantum dot evaluation method

The development of a method for evaluating the photonic usability of a semiconductor QD based on its emission spectrum is mathematically equivalent to the identification of a function $f:\,I\left( \lambda \right) \,\rightarrow \,s$ that maps a given emission spectrum $I\left( \lambda \right)$ to a rating score $s\in [0,\,1]$, with $s=1$ encoding a potentially perfect single photon source recommended for further analyses, and $s=0$ a most likely unfit candidate. As discussed in section “Semiconductor quantum dots”, given the complexity of this gauging combined with the lack of established conventions and the subjectivity involved, an analytical derivation of such a function is highly impractical. Therefore, in this paper, a data-driven regressive approach is proposed instead.

Regression analysis is the statistical estimation of a functional relationship between an independent input $u\in {\mathscr {U}}$ and a dependent output $y=f(u)\in {\mathscr {Y}}$ by minimising a loss function

1 $$\begin{aligned}  \min _\beta \;&\sum _{i=1}^{N}\,J\left( \,y^{(i)}_\text {train},\,{\hat{y}}^{(i)}_\text {train}\right) ,\; J:\,{\mathbb {R}}\,\rightarrow \,{\mathbb {R}} \\ \text {s.t.} \;&{\hat{y}}^{(i)}_\text {train}=f_\text {reg}\left( u^{(i)}_\text {train},\,\beta \right) \end{aligned}$$

over the parameter vector $\beta \in {\mathscr {B}}$ of a pre-defined regression model $f_\text {reg}:\,{\mathscr {U}}\times {\mathscr {B}}\,\rightarrow \,{\mathscr {Y}}$ using a set of available training data $D_\text {train}=\left\{ \left( u^{(i)}_\text {train},\,y^{(i)}_\text {train}=f\left( u^{(i)}_\text {train}\right) \right) \,\Big |\,i=1,\dots ,\,N\right\}$. As this involves detecting underlying patterns in the data, limiting redundancies and noise by reducing the dimension of the function space can significantly improve the overall performance of the regression. With high-dimensional data, it is therefore common practice to first compress the independent variable $u\in {\mathscr {U}}$ into a lower-dimensional feature vector $x\left( u\right) \in {\mathscr {X}}\subset {\mathscr {U}}$ from which to subsequently predict the dependent target variable $y={\hat{f}}\left( x\right)$^36,37^.

**Figure 4:**
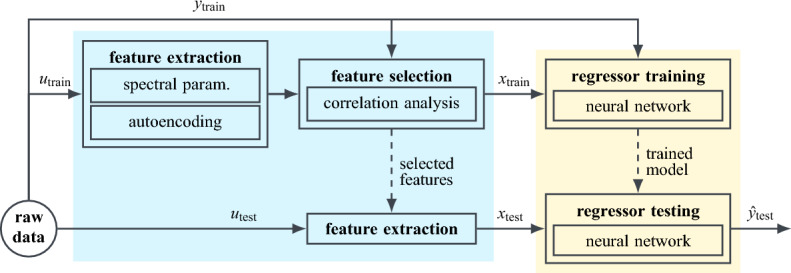
Visual abstract of a regression analysis scheme (yellow) including feature engineering (blue). In this paper, the independent input *u* corresponds to a measured QD emission spectrum $I\left( \lambda \right)$, and the dependent output *y* to the evaluation score *s* ranking the QD’s technical usability as single photon source between 0 and 1 with confidence $\sigma$.

Within the scope of this paper, this implies, a meaningful feature representation *x* is to be derived for the emission spectrum $u=I\left( \lambda \right)$ of a semiconductor QD. For this, we consider both explainable spectral parameters, extracted by conventional methods of signal processing, as well as an abstract latent representation learned by an autoencoder. A subset ${\mathscr {X}}$ of minimally redundant, but maximally relevant features, that still sufficiently accurately describes the data^38,39^, is then selected by correlation analysis and used as input of a regression model ${\hat{f}}_\text {reg}\left( x,\,\beta \right)$. Here, we propose a multivariate neural network regressor, specifically designed to not only predict a technical suitability score ${\hat{y}}_1={\hat{s}}$, but to also return a measure of confidence ${\hat{y}}_2=\sigma$ for its estimate. A visual abstract of the overall scheme is given in Fig. 4. The top half outlines the pre-processing and training, whereas the bottom half visualises the workflow for predicting the output ${\hat{y}}_\text {test}$ of some unknown test input $u_\text {test}$, with its feature representation $x_\text {test}$ being passed to the now optimised regression model. The following sections elaborate on the neural network regression analysis, highlighted in yellow, and the feature engineering, marked in blue.

### Neural network regression analysis

**Figure 5:**
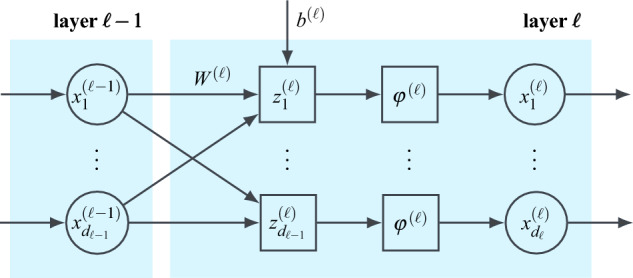
Schematic representation of a layer in a feed-forward NN.

Overall, the performance of any regression analysis is determined by the ability of the trained model to generalise, i.e. to make accurate predictions for unknown inputs $x_\text {test}$. Here, besides the quality of the training data and the numerical optimisation, the selection of the model function ${\hat{f}}_\text {reg}$ itself is key. In this regard, different regression techniques are distinguished. Most common is linear regression, which is easily implemented, but limited in its application^40^. For non-linear systems, kernel-based methods like support vector machines^41^ or Gaussian process models are widely established^42^. Lastly, artificial neural networks (NN) are a class of universal function approximators^43^ with a characteristic parameter structure of hierarchical layers intended to resemble interconnected biological neurons^44^. A fully connected feed-forward NN regression model is defined as

2 $$\begin{aligned} {\hat{f}}_\text {reg, NN}\left( x,\,\beta \right) = \Bigg [\,\underbrace{\left( z^{(1)}\circ \varphi ^{(1)}\right) }_{=x^{(1)}\left( x,\,\beta ^{(1)}\right) }\;\circ \;\dots \;\circ \underbrace{\left( z^{(L)}\circ \varphi ^{(L)}\right) }_{=x^{(L)}\left( x^{(L-1)},\,\beta ^{(L)}\right) }\Bigg ](x)\,, \end{aligned}$$

where $L\in {\mathbb {N}}$ denotes the number of layers. Besides the first layer $\ell =1$, which is passed the feature vector $x^{(0)}=x\in {\mathscr {X}}$, each layer $\ell$ is passed the output of the previous layer $x^{(\ell -1)}\in {\mathbb {R}}^{d_{\ell -1}}$ as input. The last layer, finally, returns the model predictions ${\hat{y}}=x^{(L)}$. A schematic depiction of the setup of an arbitrary layer $\ell$ is given in Fig. 5. Each layer comprises a non-linear activation function $\varphi ^{(\ell )}:\,{\mathbb {R}}\,\rightarrow \,{\mathbb {R}}$ which is applied element-wise to the output of an affine mapping

3 $$\begin{aligned}  z^{(\ell )}\left( x^{(\ell -1)},\,\beta ^{(\ell )}\right)&= W^{(\ell )}x^{(\ell -1)} + b^{(\ell )} \,,\\ \beta ^{(\ell )}&=\left\{ W^{(\ell )},\,b^{(\ell )}\right\} \end{aligned} $$

where the weight matrix $W^{(\ell )}\in {\mathbb {R}}^{d_{\ell }\times d_{\ell -1}}$ and the bias vector $b^{(\ell )}\in {\mathbb {R}}^{d_\ell }$ are the model parameters to be optimised during training. Note, that the general matrix multiplication in (3) can also be replaced by a discrete convolution with a set of learnable kernels^45^.

Compared to conventional regression models, NN regressors stand out for their huge parameter space, which is further extended by the inclusion of additional connections, shortcuts or feedbacks between the layers in more sophisticated network architectures^46,47^. Because of this, NNs are particularly good at recognising patterns in unstructured data and making generalising predictions. Accordingly, they have been applied successfully to a wide range of problems, from the calibration of biosensing systems^48^, to the evaluation of chess positions^49^ and the identification of objects in images^50^.

### Feature engineering

As discussed above, the use of properly optimised features is crucial for pattern detection in data analysis and thus for regressive function modelling, improving the overall performance and the prediction accuracy in particular. Like all spectral data, QD emission spectra are characterised by some rather self-evident features, first and foremost, the number of peaks $n_\text {peak}\in {\mathbb {N}}$, which infers how many optical transitions are excited at the same time. However, considering an ideal single photon source emits at only one specific wavelength, usually only the brightest peak with the maximum emission intensity $u_\text {max}\in {\mathbb {R}}^+$ is of interest. Its relative dominance can be quantified by the ratio of its amplitude to the height of the next larger peak, denoted by $r_\text {dom}\in {\mathbb {R}}^+$. Besides, its sharpness, best described by its full width at half maximum $w_\text {FWHM}\in {\mathbb {R}}^+$, and its minimum distance $d_\text {min}\in {\mathbb {R}}^+$ to neighbouring peaks determine the feasibility of isolating the corresponding level transition for single photon generation. Note, that at this stage, the exact emission wavelength is of secondary importance and will hence not be taken into account.

**Figure 6:**
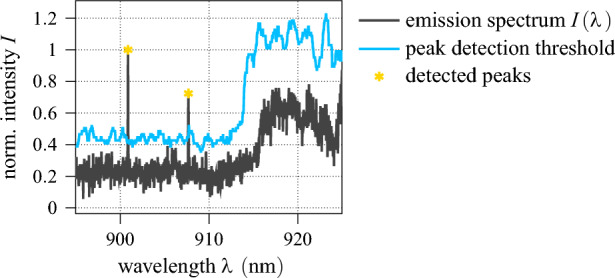
Working principle of the OS-CFAR peak detection algorithm^51^. Data points above the adaptive threshold are identified as spectral peaks.

All of the mentioned features represent explainable parameters that can be extracted from the data using conventional methods of signal processing. Here, we employ the *Ordered Statistics Constant False Alarm Rate* (OS-CFAR) peak detection algorithm, which is commonly used in radar technology, as it is capable of adapting the detection threshold to the surrounding noise baseline^51^. As showcased in Fig. 6, this prevents spectral broadband features to be identified as a collection of subsequent individual peaks. Once the peaks have been localised within the spectrum, the algebraic computation of the corresponding feature values is straightforward.

These features, however, are not necessarily sufficient to describe every aspect of an emission spectrum and to fully evaluate QDs in regard to their suitability as single photon sources. Therefore, additional abstract features are extracted using a so-called autoencoder, an unsupervised machine learning technique for non-linear dimensionality reduction^52^ and representation learning^53^.

**Figure 7:**
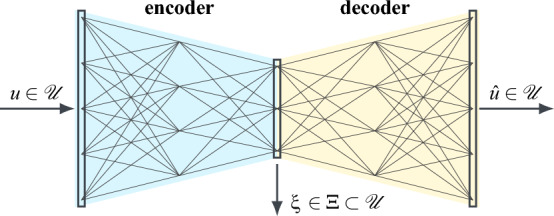
Schematic representation of an autoencoder NN. The input vector *u* is first encoded into a low-dimensional latent feature representation $\xi$, which is subsequently decoded again to produce an estimate ${\hat{u}}$ of the input.

At its core, an autoencoder is a NN regression model estimating the identity function that projects an independent variable $u\in {\mathscr {U}}$ onto itself. However, the network is set up as such, that the output of one intermediate layer is of reduced dimension^54,55^. As can be seen in Fig. 7, this implies, the information contained in the data vector *u* is first encoded into a lower-dimensional latent feature vector $\xi \in \Xi \subset {\mathscr {U}}$ and subsequently decoded again, in order to produce a reconstruction ${\hat{u}}\in {\mathscr {U}}$ of the original input. Training the network by minimising the reconstruction error $e_\text {recon}=\Vert u-{\hat{u}}\Vert _2\in {\mathbb {R}}$ requires as little information as possible to be lost when propagating the data vector through the network. Hence, the latent representation $\xi$ is automatically optimised as well and can subsequently be extracted by evaluating only the encoder part of the network (blue). Note, that, while highly informative, the features derived this way are not necessarily explainable or unique. The residual reconstruction error $e_\text {recon}$, meanwhile, provides a measure for the loss of information and thus for deviations from learned patterns and regularities. It is therefore often considered in fault detection, for instance to catch sensor or actuator errors^56,57^. In the context of this paper, it is exploited to quantify noise and spectral oddities, like the broadband feature in Fig. 6.

As outlined in section “Quantum dot evaluation method”, subsequently, a set of minimally redundant, but maximally relevant features is to be selected to be used as input *x* for the NN regression model estimating a QD’s viability as single photon source. The results hereof are presented in the next section.

## Results

**Figure 8:**
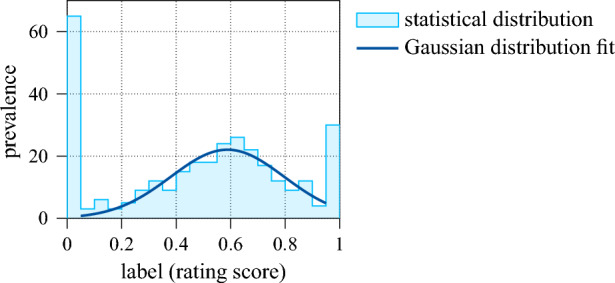
Rating score distribution in the labelled dataset as histogram.

In this section, the proposed evaluation algorithm is implemented and validated. For this, we consider single layer ${\text {InAs/GaAs}}$ semiconductor QDs within a n–i–n diode structure. The QDs are located in the vertical antinode of a planar cavity formed by a bottom distributed Bragg reflector (DBR) and a lower reflectivity top DBR. More information on the samples can be found in^22^, alongside comprehensive optical and quantum optical characterisations. Using an above-band excitation laser close to saturation, a dataset of ${25\,000}$ emission spectra is recorded in a spectral range of ${30}\,{\textrm{nm}}$ in ${1024}\,{\textrm{px}}$, thus giving an input dimension of $\dim \,{\mathscr {U}}={1024}$. Moreover, a total of 300 spectra is labelled redundantly by a team of seven experienced experts in the field, i.e. personal biases are reduced to a minimum by the assignment of a score average *s* between 0 and 1 that rates the viability of the emitting QD as source of single, indistinguishable photons with isolated, bright emission lines and low background. An approximate representation of the score distribution is given by the histogram in Fig. 8.

Excluding the marginal extrema, the distribution is roughly Gaussian (dark blue curve). Lastly, to augment the data, each sample is spectrally shifted twice by a random number of pixels. As this does not affect any spectral properties qualitatively, the size of both the labelled and unlabelled dataset is increased by a factor of three, which benefits the training of the various machine learning models. For this, as is common practice, 80% of the data is used, with the remaining 20% being retained for testing and to track possible overfitting. Note, that this split is applied to both the labelled and the unlabelled dataset.

In the following, first, the results of the autoencoder feature extraction are presented, then, a suitable subset of features is selected by correlation analysis, and eventually, the performance of the rating score prediction by NN regression is showcased.

### Autoencoder representation learning

For the derivation of an abstract feature representation for QD emission spectra, in this paper, an autoencoder with latent space dimension $\dim \,\Xi ={16}$ is proposed. This is the result of a hyper-parameter optimisation and represents a trade-off between the loss of too much information in case the latent space dimension is too small, and a reduced correlation of the autoencoder states with the rating score in case the latent space dimension is too large. Taking normalised input data with $\Vert u\Vert _\text {max}=1$, the encoder part handles the feature learning and the dimensionality reduction. For the former, typically, deep convolutional NNs are employed, which excel at pattern detection, but potentially suffer from vanishing or exploding gradients during optimisation^58^. In this regard, residual blocks, i.e. sequences of two convolutional layers with a skip connection, offer some numeric benefits, as any derivation yields at least an identity matrix. Moreover, the mapping of linear relations is facilitated^59,60^.

For the dimensionality reduction, in return, max pooling is state of the art. This is a downsampling technique, where the output of a convolutional layer is divided into blocks of equal size, with only the maximum value of each block being propagated further. Since this way the most dominant entries are retained, the overall performance of the network is not significantly impaired. On the contrary, max pooling introduces a certain degree of translational invariance and improves the computational efficiency of the network^61,62^.

**Figure 9:**
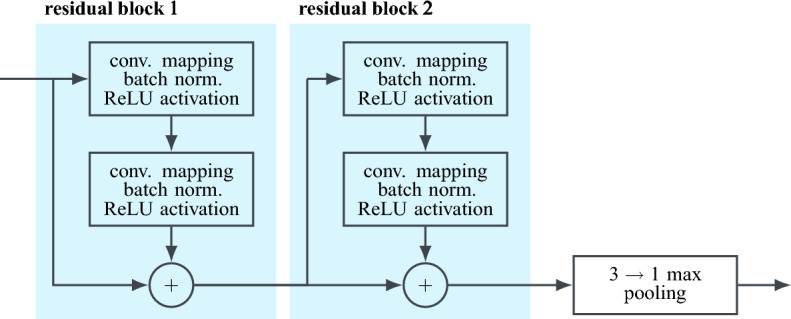
Schematic visualisation of a residual unit, consisting of two residual blocks followed by max pooling.

Here, the encoder is set up as a series of four residual units, each consisting of two residual blocks followed by max pooling (ref. Fig. 9). As denoted, the results of the convolutional mappings are batch normalised before being passed to the rectified linear unit (ReLU) activation function^63^

4 $$\begin{aligned} \varphi _{\,\text {ReLU}}(x)=\max \left( 0,\,x\right) . \end{aligned}$$

**Figure 10:**
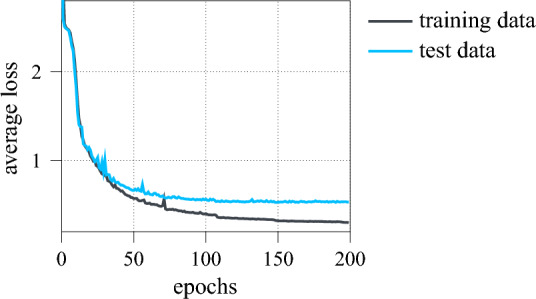
Average training and test loss of the autoencoder during training.

**Figure 11:**
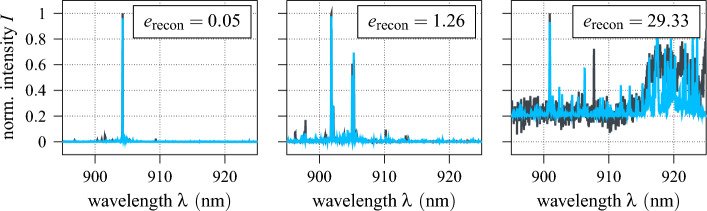
Autoencoder reconstruction of three model QD emission spectra.

In each residual unit, the four convolutional layers have the same structure and hyper-parameters (output shape, kernel size, stride, padding), whereas a max pooling dimensionality reduction by factor three is adopted throughout. Lastly, the compact latent representation $\xi \in \Xi ,\,\dim \,\Xi ={16}$ is produced by a fully connected feed-forward layer. For the subsequent recovery of the input vector and the associated increase in dimensionality, the decoder comprises six sequential transposed convolutional layers^64^. Since the input data is normalised, the sigmoid activation function

5 $$\begin{aligned} \varphi _{\,\text {sigmoid}}\left( x\right) =\frac{1}{1+e^{-x}}\in [0,\,1] \end{aligned}$$

is used here to constrain the output value range such that $\Vert {\hat{u}}\Vert _\text {max}\le 1$. A summary of the autoencoder’s complete architecture is given in Table 1. Overall, the autoencoder has 233313 training parameters, which are optimised with respect to the squared $\ell ^2$-norm using the computationally efficient ADAM algorithm with learning rate scheduling^65^ and a batch size of 512. As the autoencoder training is unsupervised, the unlabelled dataset is used for it. Fig. 10 displays the training and test learning curve of the autoencoder over 200 epochs of optimisation. Clearly, both decay approximately exponentially towards 0, indicating that the autoencoder’s learnt latent representation is optimised without significant overfitting. The performance of the fully trained autoencoder is further showcased in Fig. 11, where the reconstructions of the three model spectra from Fig. 3 are shown. Note, that these are part of the test data and therefore not previously known. As can be seen, the first two spectra are recovered reasonably well, with all major peaks captured and the reconstruction errors correspondingly low. In contrast, the autoencoder struggles to reconstruct the third spectrum, as both the worse signal-to-noise ratio and the spectral broadband feature constitute sever deviations from learnt patterns and regularities. As discussed in section “Feature engineering”, the reconstruction error $e_\text {recon}$ is considered as feature precisely to take such cases into account. On the other hand, no physical meaning could be inferred for the autoencoder’s latent states.

**Table 1:** Autoencoder architecture summary.

Layer		Output shape	Parameter
Residual unit	Kernel 3, stride 3, padding 1	$4 \times 341$	248
Residual unit	Kernel 3, stride 3, padding 1	$16 \times 113$	3344
Residual unit	Kernel 3, stride 3, padding 1	$32 \times 37$	13,216
Residual unit	Kernel 3, stride 3, padding 1	$64 \times 12$	52,032
Flatten		$1 \times 768$	–
Linear feed-forward		$1 \times 16 = \dim \,\Xi$	12,304
Linear feed-forward		$1 \times 4608$	78,336
Reshape		$128 \times 36$	–
Transposed conv. layer	Kernel 7, stride 3	$64 \times 112$	57,664
Transposed conv. layer	Kernel 6, stride 3	$32 \times 339$	12,448
Transposed conv. layer	Kernel 6, stride 3	$16 \times {1020}$	3152
Transposed conv. layer	Kernel 3, stride 1, padding 1	$8 \times {1020}$	424
Transposed conv. layer	Kernel 3, stride 1, padding 1	$4 \times {1020}$	116
Transposed conv. layer	Kernel 5, stride 1	$1 \times {1024} = \dim \,{\mathscr {U}}$	29

### Feature selection

**Figure 12:**
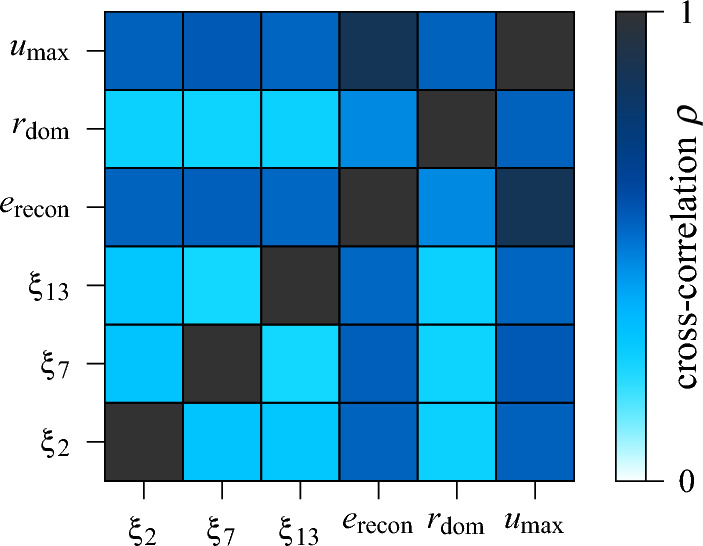
Absolute of Spearman’s rank cross-correlation coefficient between features in (7).

Combining the autoencoder’s latent representation $\xi$ and reconstruction error $e_\text {recon}$ with the aforementioned characteristic spectral parameters, overall, a set of 22 features

6 $$\begin{aligned} \Big \{\xi _1,\,\dots ,\,\xi _{16},\,e_\text {recon},\,n_\text {peak},\,r_\text {dom},\,u_\text {max},\,w_\text {FWHM},\,d_\text {min}\Big \} \end{aligned}$$

can be extracted from each QD emission spectrum $u=I\left( \lambda \right)$. These are, however, neither inherently independent, nor necessarily impacting the suitability evaluation subject to this paper. Therefore, two correlation studies are performed to select a subset of minimally redundant, but maximally relevant features to be used as input vector *x* for the NN regression model. For both, the absolute of Spearman’s rank correlation coefficient $\rho \in [-1,\,1]$ is used, as it is not limited to linear relationships, but rather measures monotonicity^66^. First, using the available labelled training data, the correlation between each feature and the rating score is computed. The results hereof are listed in Table 2. In particular, the reconstruction error $e_\text {recon}$ and the maximum emission intensity $u_\text {max}$ stand out for their strong correlation of $\rho >{0.9}$ with the target value. Subsequently, only features with a correlation coefficient $\rho >{0.6}$ are retained, which reduces the set of features under consideration to

7 $$\begin{aligned} \Big \{\xi _2,\,\xi _7,\,\xi _{13},\,e_\text {recon},\,r_\text {dom},\,u_\text {max}\Big \}, \end{aligned}$$

where the significant amount of latent features justifies the use of the autoencoder. The remaining features are subject to a cross-correlation analysis. The results are visualised in Fig. 12. Clearly, the reconstruction error $e_\text {recon}$ and the maximum emission intensity $u_\text {max}$ are also correlated comparatively strongly with each other. Furthermore, both show a moderate cross-correlation with the remaining latent features $\xi _{2,\,7,\,13}$. Since for the reconstruction error this can be attributed to the shared origin, i.e. the autoencoder, the maximum emission intensity is omitted in order to limit redundancies. This leaves five features, that combined form the input vector

8 $$\begin{aligned} x=\begin{bmatrix} \xi _2&\xi _7&\xi _{13}&e_\text {recon}&r_\text {dom} \end{bmatrix}^\top \in {\mathscr {X}}\, ,\; \dim \,{\mathscr {X}}=5 \end{aligned}$$

of the NN regression model estimating a QD’s viability as single photon source. The comparatively high relevance and impact of the reconstruction error $e_\text {recon}$ is revisited in section “Evaluation score prediction”.

**Table 2:** Absolute Spearman correlation between each feature and the rating score.

$\xi _{1}$	${\xi _{2}}$	$\xi _{3}$	$\xi _{4}$	$\xi _{5}$	$\xi _{6}$	${\xi _{7}}$	$\xi _{8}$	$\xi _{9}$	$\xi _{10}$	$\xi _{11}$
0.438	$\pmb {{0.647}}$	0.198	0.106	0.509	0.391	$\pmb {{0.694}}$	0.094	0.244	0.376	0.276

$\xi _{12}$	${\xi _{13}}$	$\xi _{14}$	$\xi _{15}$	$\xi _{16}$	${e_\text {recon}}$	$n_\text {peak}$	${r_\text {dom}}$	${u_\text {max}}$	$w_\text {FWHM}$	$d_\text {min}$
0.208	$\pmb {{0.637}}$	0.588	0.064	0.559	$\pmb {{0.921}}$	0.488	$\pmb {{0.648}}$	$\pmb {{0.908}}$	0.398	0.379

Significant values with |ρ| > 0.6 are in bold.

### Evaluation score prediction

The last building block of the proposed QD evaluation scheme is the NN regression model. Considering the goal is to replicate an expert’s experienced based decision process with its inherent subjectivity, the network is set up as such, that not only a rating score prediction ${\hat{s}}\in [0,\,1]$ is returned, but also a measure of confidence $\sigma \in {\mathbb {R}}$ for it. To do so, using the training data, the Gaussian negative log-likelihood loss function

9 $$\begin{aligned} &J=\ln (\sqrt{2\,\pi \,\sigma ^2})+\frac{1}{2}\,\frac{\left\| s-{\hat{s}}\right\| ^2}{\sigma ^2}\\&\text {s.t.}\;\begin{bmatrix} {\hat{s}} \\ \sigma \end{bmatrix}={\hat{f}}_\text {reg, NN}\left( x,\,\beta \right) . \end{aligned}$$

is minimised over $\beta$ for the multivariate NN regression model ${\hat{f}}_\text {reg, NN}$ with vector-valued output. For accurate predictions, this causes the optimiser to drive $\sigma \,\rightarrow \,0$, whereas for inaccurate predictions, $\sigma$ must inevitably increase for the second summand to be minimised. Note, that this is accomplished without supervision. Given that (9) is the negative natural logarithm of a normal distribution, $\sigma$ can be interpreted as standard deviation of the prediction and is hence referred to as such^67^.

**Figure 13:**
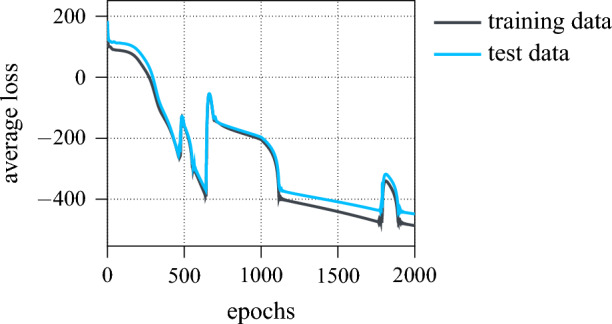
Average training and test loss of the NN regression model during training.

The regression model itself is designed as a fully connected feed-forward NN with four layers, using the sigmoid activation function (4) throughout to constrain the predictions to ${\hat{s}}\in [0,\,1]$. Table 3 provides further details regarding the architecture of the network. As before, the ADAM optimisation algorithm is employed with a batch size of 64 and the resulting training and test learning curves over 2000 epochs are given in Fig. 13. Despite several outliers, both curves clearly decay and the network is accordingly optimised with negligible overfitting. In fact, considering the widespread $\text {R}^2$ score as accuracy metric for regression analysis^68^, a training score of 96%, and a test score of 95% is achieved. For reference, taking the reconstruction error, i.e. the strongest feature, as single input for the regression model such that $x=e_\text {recon}$, yields a $\text {R}^2$ test score of 84%. Considering the reconstruction error primarily quantifies noise, this implies, that differentiating candidate QD spectra solely with respect to the signal-to-noise ratio would lead to significantly worse results.

**Table 3:** NN regression model architecture summary.

Layer	Output shape	Parameter
linear feed-forward	$1 \times 20$	120
linear feed-forward	$1 \times 20$	420
linear feed-forward	$1 \times 20$	420
linear feed-forward	$1 \times 2$	42

**Figure 14:**
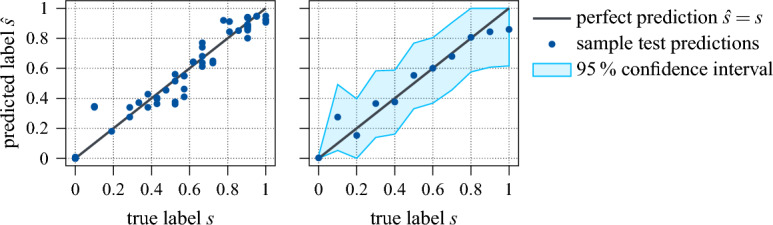
Prediction of the NN regression model, plotted as function of the actual label. Left: Predictions for one test batch. Right: Predictions for test samples with equidistant true labels (increment 0.1).

The general performance of the fully trained network is plotted in Fig. 14. Here, the predicted rating scores are plotted over the ground truth, on the left for one entire test batch, on the right for randomly selected test samples of equal label increment with the estimated 95% confidence interval ($\pm {1.96}\,{\sigma }$). The identity represents perfect estimation. As can be seen, the predictions are overall quite accurate with a mean absolute deviation of 0.05 corresponding to a relative error of 5%. In particular, the assignment of rating score zero is both very precise and with high confidence, whereas low, but non-zero ratings are slightly overestimated. Nonetheless, the ground truth always lies within the estimated 95% confidence interval and the predictions are therefore throughout reasonably good.

**Figure 15:**
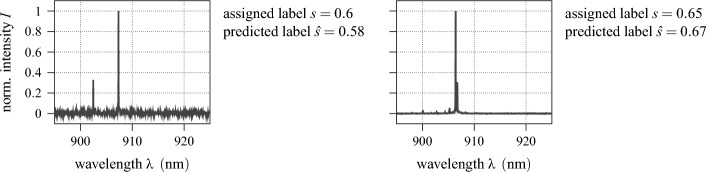
Additional QD emission spectra examples, drawn from the labelled test dataset.

To illustratively showcase the practical use of the proposed method, two example spectra are given in Fig. 15, alongside their respective label predictions. Both QDs are, solely based on their emission spectrum, to be considered as candidates of mediocre quality. For the model spectra given in Fig. 3, meanwhile, rating scores of ${\hat{s}}=\left\{ 0.95,\,0.45,\,0.01\right\}$ are predicted from left to right, whereas they had been labelled $s=\left\{ 0.97,\,0.5,\,0.1\right\}$, respectively. As can be seen, both the expert as well as the proposed expert system agree on their evaluation, highlighting the potential for automatisation in this field.

## Discussion and outlook

The main objective of this paper was the development of a method to automatically evaluate the viability of a semiconductor QD as single photon source based on its emission spectrum. For this, combining spectral analysis and an autoencoder, a suitable feature representation for QD emission spectra is derived and a NN regression model is trained on a given set of expert labelled data. Overall, the proposed solution achieves highly convincing results by reliably predicting accurate ratings for unknown test inputs. Embedding the evaluation algorithm in a user application and establishing a required minimum rating enables the automation of the manual pre-selection of candidate QDs for further analyses. This does not only significantly reduce processing times, but also introduces a certain degree of objectivity and comparability. Overall, this work showcases how machine learning can support and benefit the ongoing development of quantum technologies by solving practical challenges.

However, several aspects are to be pointed out in this context. First, in this paper, a regressive rather than a classification based approach is employed. This has the advantage, that the cut-off score can be chosen freely to render the selection more conservative or more radical. In fact, it can even be defined as a function of the estimated measure of confidence of the rating prediction. Secondly, note, that the data used here to train the regression model was labelled by a team of experts to eliminate any personal bias. In practice, however, different experts work on different topics and therefore have different spectral requirements. In particular, the distinction between exciton, biexciton and trion excitation is technically highly relevant. This can be accounted for by optimising the network only with regard to the labels assigned by one expert. In this case, the trained model will replicate their personal assessment and will be tuned to their application scenario. Since only a comparatively small dataset is required to be re-labelled and the cross-training of the prediction model is of low computational effort, adapting the proposed evaluation method is considerably more efficient than adjusting a rating system not based on machine learning, for which a plethora of decision variables and threshold values would have to be fine-tuned. Note, that the demand for QDs of one specific emission wavelength can be met by simply filtering the evaluated and selected spectra accordingly, which is why this parameter was not included as feature in the analysis. Finally, it should be mentioned, that while this work focusses on self-assembled QDs grown in the Stranski–Krastanow mode by molecular beam epitaxy, comparable challenges arise with other fabrication methods as well. However, since the solution proposed here is transferable, the same approach can be adopted for each fabrication method, material composition and photonic structure.

In the long-term, the framework presented in this paper is to be expanded to a fully automated evaluation tool for semiconductor QDs, capable of taking into account not only emission spectra, but also further measurements and custom requirements, in order to streamline and support the synthesis of high quality single photon sources.

## Data Availability

Both code and data will be made available by the corresponding author upon reasonable request.
